# Influence of High Hemoglobin-Oxygen Affinity on Humans During Hypoxia

**DOI:** 10.3389/fphys.2021.763933

**Published:** 2022-01-14

**Authors:** Kevin L. Webb, Paolo B. Dominelli, Sarah E. Baker, Stephen A. Klassen, Michael J. Joyner, Jonathon W. Senefeld, Chad C. Wiggins

**Affiliations:** ^1^Department of Anesthesiology and Perioperative Medicine, Mayo Clinic, Rochester, MN, United States; ^2^Department of Kinesiology, University of Waterloo, Waterloo, ON, Canada; ^3^Department of Kinesiology, Brock University, St. Catharines, ON, Canada

**Keywords:** altitude acclimatization, high-altitude, oxygen transport, exercise, VO_2max_ (maximal oxygen uptake), high affinity hemoglobin (Hb)

## Abstract

Humans elicit a robust series of physiological responses to maintain adequate oxygen delivery during hypoxia, including a transient reduction in hemoglobin-oxygen (Hb-O_2_) affinity. However, high Hb-O_2_ affinity has been identified as a beneficial adaptation in several species that have been exposed to high altitude for generations. The observed differences in Hb-O_2_ affinity between humans and species adapted to high altitude pose a central question: is higher or lower Hb-O_2_ affinity in humans more advantageous when O_2_ availability is limited? Humans with genetic mutations in hemoglobin structure resulting in high Hb-O_2_ affinity have shown attenuated cardiorespiratory adjustments during hypoxia both at rest and during exercise, providing unique insight into this central question. Therefore, the purpose of this review is to examine the influence of high Hb-O_2_ affinity during hypoxia through comparison of cardiovascular and respiratory adjustments elicited by humans with high Hb-O_2_ affinity compared to those with normal Hb-O_2_ affinity.

## Introduction

Currently, there is ongoing debate about the advantages of higher or lower hemoglobin-oxygen (Hb-O_2_) affinity in humans, particularly during hypoxia (Dempsey, 2020). A decrease in Hb-O_2_ affinity is often observed among humans during acclimatization to altitudes ranging from 2500 to 4500 m, presumably to facilitate O_2_ off-loading and protect against tissue hypoxia (Hall et al., 1936; Aste-Salazar and Hurtado, 1944; Lenfant and Sullivan, 1971). In contrast, several animal species adapted to high-altitude environments display a higher Hb-O_2_ affinity compared to that of low-land counterparts (Bartels et al., 1963; Monge and Leon-Velarde, 1991; Weber et al., 1993; Scott and Milsom, 2007; Storz et al., 2010; Storz, 2016; Natarajan et al., 2018). These divergent observations lead to the central question of this review, is higher or lower Hb-O_2_ affinity more advantageous for humans during hypoxia?

Humans rely on a continuous supply of O_2_ for metabolism. Oxygen binds to hemoglobin in the lungs and travels through the large arteries, arterioles, and finally the small capillaries supplying peripheral tissue (Scholander, 1960). Although *in vitro* Hb-O_2_ affinity is characterized by a single curve or metric (e.g., P_50_, as described below), the *in vivo* Hb-O_2_ affinity cannot be described as simply. Within the vasculature, alterations of modulatory factors such as temperature, pH, and the concentration of carbon dioxide (CO_2_) lead to transient changes in Hb-O_2_ affinity during circulatory transit, which directly impact O_2_ loading at the lung and O_2_ off-loading in peripheral tissue (Jensen, 2004; Winslow, 2007). Changes in Hb-O_2_ affinity can be transient or chronic due to a variety of conditions such as genetic mutations, disease, altitude acclimatization, or age (Woodson et al., 1970; Humpeler and Amor, 1973; Versmold et al., 1973; Winslow, 2007). For example, evidence suggests that some groups of humans native to high altitude have a greater Hb-O_2_ affinity than sea-level residents (Simonson et al., 2014; Li et al., 2018). Although the mechanisms underlying the adaptive increase of Hb-O_2_ affinity among high-altitude natives are not well understood, a number of genetic mutations in hemoglobin structure that contribute to a systemic increase in Hb-O_2_ affinity in humans have been identified (Mangin, 2017), predominantly among low-altitude residents. Humans with mutations resulting in high Hb-O_2_ affinity may provide unique insight to the ongoing debate regarding the advantages and disadvantages of high Hb-O_2_ affinity during hypoxia. Past investigations of the cardiorespiratory adjustments to hypoxic exposure at rest and during exercise suggest that high Hb-O_2_ affinity may provide better maintenance of O_2_ delivery and utilization in humans. Therefore, the purpose of this review is to highlight the potential advantages and disadvantages of high Hb-O_2_ affinity in humans during hypoxia through examination of cardiovascular and respiratory adjustments at rest and during exercise.

To address the central question of this review, we examine available studies reporting cardiovascular or respiratory adjustments to hypoxia at rest or during exercise in humans with genetic mutations resulting in high Hb-O_2_ affinity. To avoid confounding factors that may alter cardiovascular and respiratory responses, we excluded studies in which these individuals have recently undergone venesection. Studies fitting these criteria can be found in Table 1, including participant characteristics and experimental design. To clearly denote the “severity” of hypoxia within the discussion, we define low altitude as <2500 m, high altitude as >2500 m, and extreme altitude as >7000 m.

**TABLE 1:** Studies examining cardiorespiratory adjustments during normoxia or hypoxia in humans with high Hb-O_2_ affinity.

Study	Age (years)	Sex (*n*)	Hb type	P_50_ (mmHg)	[Hb] (g/dL)	Hct (%)	Study design
Hebbel et al., 1977	12	1M	Hb Andrew-Minneapolis	17	16	*NR*	Hypoxic ventilatory response (F_*i*_O_2_ = 0.13, ∼3800 m)
	18	1F	Hb Andrew-Minneapolis	17	17	*NR*	

Hebbel et al., 1978	12	1M	Hb Andrew-Minneapolis	17	17	48	High-altitude acclimatization (∼3100 m) and graded cycling to exhaustion
	18	1F	Hb Andrew-Minneapolis	17	17	50	

Rossoff et al., 1980	25	2M	Hb Rainier	12	*NR*	*NR*	Hypoxic ventilatory response (F_*i*_O_2_ = 0.14, ∼3300 m)

Wranne et al., 1983	30	1M	*NR*	14	19	55	Normoxic submaximal cycling
	31	1M	*NR*	14	18	54	

Länsimies et al., 1985	38 (14)	5M	Hb Linköping	16 (0.4)	19 (1)	*NR*	Normoxic graded cycling to exhaustion
	32 (8)	5F	Hb Linköping	17 (0.5)	16 (4)	*NR*	

Dominelli et al., 2019	45 (8)	3M	Hb Malmö	15 (0.2)	21 (1)	63 (3)	Hypoxic ventilatory response (F_*i*_O_2_ = 0.14, ∼3300 m)
	43 (15)	6F	Hb Malmö (*n* = 5), Hb San Diego (*n* = 1)	16 (1.1)	19 (1)	55 (3)	

Dominelli et al., 2020	45 (8)	3M	Hb Malmö	15 (0.2)	21 (1)	63 (3)	Normoxic and normobaric hypoxic (F_*i*_O_2_ = 0.15, ∼2600 m) graded cycling to exhaustion
	31 (9)	8F	Hb Malmö (*n* = 7), Hb San Diego (*n* = 1)	16 (0.9)	18 (1)	54 (2)	

*The fraction of inspired O_2_ and associated elevation are provided under study design. Abbreviations: M, male; F, female; Hb, hemoglobin; P_50_, the P_O_2__ at which 50% of hemoglobin is saturated with O_2_; Hb, hemoglobin; Hct, hematocrit; F_i_O_2_, fraction of inspired O_2;_ NR, not reported. Numbers within parentheses throughout the table indicate standard deviations.*

## Foundational Concepts

**FIGURE 1:**
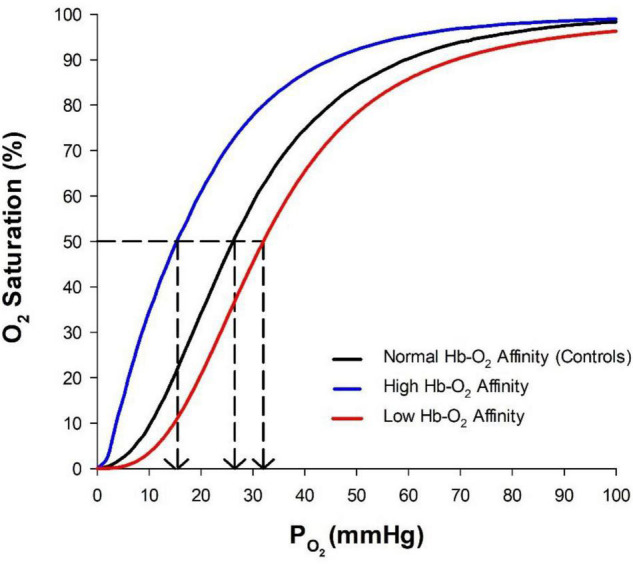
Oxygen dissociation curve showing normal hemoglobin-O_2_ (Hb-O_2_) affinity (P_50_ ∼26 mmHg), high Hb-O_2_ affinity (P_50_ ∼16 mmHg), and low Hb-O_2_ affinity (P_50_ ∼32 mmHg). The P_50_, denoted by the dashed lines, is defined as the P_*O*_2__ at which 50% of hemoglobin is saturated with O_2_.

Hemoglobin-oxygen affinity is largely determined by the structure of hemoglobin and modulated by a variety of factors within the vasculature [temperature, pH, CO_2_, 2,3-diphosphoglycerate (2,3-DPG), organic phosphates, chloride ions (Cl^–^), etc.] (Mairbaurl et al., 1993). The relationship between the partial pressure of O_2_ (P_*O*_2__) and O_2_ saturation can be described by the O_2_ dissociation curve (Figure 1). One common metric to quantify Hb-O_2_ affinity is P_50_, defined as the P_*O*_2__ at which 50% of hemoglobin is saturated with O_2_. A lower P_50_ corresponds to a higher Hb-O_2_ binding affinity or a “left-shifted” O_2_ dissociation curve. On the other hand, a higher P_50_ corresponds to a lower Hb-O_2_ binding affinity and a “right-shifted” O_2_ dissociation curve. In addition to P_50_, the Hill coefficient is often used to describe the curvature of the O_2_ dissociation curve (Endrenyi et al., 1975; Piiper, 1992; Riggs, 1998). However, describing the O_2_ dissociation curve with the P_50_ and the Hill coefficient presents some limitations. Experimentally, the P_50_ and Hill coefficient are commonly determined using *in vitro* standardized environmental conditions [pH ∼7.4, partial pressure of CO_2_ (P_*CO*_2__) ∼40 mmHg, and temperature ∼37°C], which does not account for transient changes in the *in vivo* modulation of Hb-O_2_ affinity during circulatory transit (Braumann et al., 1982). Therefore, there is not “one” O_2_ dissociation curve because the binding affinity and cooperativity of hemoglobin vary throughout the vasculature. Nevertheless, standardized measurements of P_50_ and the Hill coefficient allow general inter-individual comparisons of Hb-O_2_ affinity, but do not account for *in vivo* modulation of Hb-O_2_ affinity.

Changes in Hb-O_2_ affinity throughout the vasculature optimize both O_2_ loading in the lungs and O_2_ off-loading to peripheral tissue. For example, byproducts of metabolism (increased temperature, increased CO_2_, and lower pH) contribute to a localized decrease in Hb-O_2_ affinity in exercising muscle, thereby promoting O_2_ off-loading and utilization (Böning et al., 1975). Furthermore, a lower temperature and increased pH within the lung result in a localized increase in Hb-O_2_ affinity and improved O_2_ loading (Mairbäurl, 2013). Alternatively, long-term regulation of modulatory factors or alterations in the structure of hemoglobin can lead to systemic wide changes in Hb-O_2_ affinity. For instance, hypoxia increases 2,3-DPG concentration (due to increased glycolytic activity) in red blood cells contributing to a systemic decrease of Hb-O_2_ affinity (Lenfant et al., 1968). Standard teaching supports that a decrease in Hb-O_2_ affinity facilitates O_2_ off-loading during hypoxia (Hall et al., 1936; Aste-Salazar and Hurtado, 1944). Yet, the systemic decrease in Hb-O_2_ affinity would compromise O_2_ loading in the lung, particularly when O_2_ availability is limited during hypoxia. At higher altitudes, a decrease in Hb-O_2_ affinity would be even more disadvantageous and further compromised O_2_ loading would likely impede peripheral O_2_ delivery. Conversely, an increase in Hb-O_2_ affinity during hypoxia promotes O_2_ loading within the lungs and mitigates reductions in arterial O_2_ saturation (Eaton et al., 1974; Yalcin and Cabrales, 2012). In addition, the advantages conferred by increased Hb-O_2_ affinity are augmented at higher altitudes, outweighing potential limitations in O_2_ off-loading (Eaton et al., 1974). Therefore, homeostatic maintenance of O_2_ delivery and utilization during hypoxia is contingent on the balance between O_2_ loading in the lungs and O_2_ off-loading in the periphery, both of which are largely determined by the Hb-O_2_ affinity. Additional discussion of hemoglobin structure and the regulation of Hb-O_2_ affinity is presented below (see section “Hemoglobin-Oxygen Affinity”).

### Hemoglobin-Oxygen Affinity

Hemoglobin is a tetramer consisting of two α-subunits and two β-subunits (Coates, 1975). Each subunit contains a heme group that is capable of reversibly binding O_2_ (Perutz, 1963). When hemoglobin is fully saturated four O_2_ molecules are bound independently to each of the four subunits of the hemoglobin molecule. Hemoglobin undergoes a conformational shift with each O_2_ molecule that binds, existing in a T (tense) state when deoxygenated and a R (relaxed) state when oxygenated, commonly described by a two-state model (Monod et al., 1965). As each individual subunit becomes oxygenated, a conformational shift further increases binding affinity for O_2_ (Mihailescu and Russu, 2001). This cooperativity in O_2_ binding to hemoglobin gives rise to the sigmoidal shape of the O_2_ dissociation curve (Figure 1).

Hemoglobin is subject to allosteric regulation by multiple ligands. Most notably, higher concentrations of H^+^ and CO_2_ reduce Hb-O_2_ affinity (Riggs, 1960; Ho and Russu, 1987). This pH dependent change of Hb-O_2_ affinity is termed the Bohr effect (Bohr et al., 1904). Hb-O_2_ affinity is also reduced at higher temperatures (Weber and Campbell, 2011). For example, an increase of temperature from 37 to 40°C raises P_50_ from normal values of ∼27 to 30 mmHg (Hlastala et al., 1977). Additionally, the magnitude of the Bohr effect is greater at higher temperatures, further promoting O_2_ off-loading from hemoglobin (Hlastala et al., 1977). The cooperative effect of a more acidic environment along with higher temperatures, as occurs during rigorous exercise, significantly reduces Hb-O_2_ affinity such that P_50_ may increase up to ∼40 mmHg within the vasculature (Thomson et al., 1974). In the case of severe respiratory alkalosis, a fivefold increase in minute ventilation may reduce arterial P_*CO*_2__ from normal values of ∼40 mmHg to as low as 7 mmHg and blood pH may exceed 7.7 (Houston et al., 1987; West, 2006). At extreme altitudes, *in vivo* P_50_ may be reduced to less than 20 mmHg due to changes in blood P_*CO*_2__ and pH (West, 1984; Winslow et al., 1984).

The erythrocytic concentrations of 2,3-DPG and Cl^–^ are associated with more long-term modulation of Hb-O_2_ affinity. In effect, 2,3-DPG and Cl^–^ bind to deoxygenated hemoglobin and stabilize the T state, reducing Hb-O_2_ affinity (Benesch et al., 1967; Brewer, 1974). 2,3-DPG reduces Hb-O_2_ affinity and increases the cooperativity of hemoglobin, which “right-shifts” the O_2_ dissociation curve and steepens the slope (Tyuma et al., 1971). In addition, an influx of Cl^–^ into the red blood cell, coupled to the outward transport of bicarbonate, reduces Hb-O_2_ affinity (Wieth et al., 1982; Perutz et al., 1994; Prange et al., 2001). These ligands elicit independent effects on Hb-O_2_ affinity, and complex *in vivo* interactions between ligands give rise to the physiological P_50_ of hemoglobin. For example, 2,3-DPG and Cl^–^ compete for binding to hemoglobin and the effect 2,3-DPG on Hb-O_2_ affinity disappears at high concentrations of Cl^–^ (Imai, 1982). In addition, the Bohr effect is more pronounced at greater concentrations of 2,3-DPG (Bauer, 1969). The interested reader may consult other sources for more detailed discussions on modulation of Hb-O_2_ affinity (Antonini and Brunori, 1970; Mairbaurl and Weber, 2012).

The severity and duration of hypoxia is an important factor when considering *in vivo* modulation of Hb-O_2_ affinity in humans. During sojourns to altitudes of ∼4500 m or less, humans demonstrate a reduced Hb-O_2_ affinity due to elevated production of 2,3-DPG (Lenfant et al., 1968). At these elevations, hyperventilation reduces blood P_*CO*_2__ and potentially results in respiratory alkalosis (Dempsey and Forster, 1982). However, renal compensation leads to the excretion of excess bicarbonate and conservation of H^+^, normalizing blood pH to sea-level values after a few days at high altitude (Goldfarb-Rumyantzev and Alper, 2014; Bird et al., 2021). At higher elevations (4500–5400 m), hyperventilation becomes so pronounced that renal compensation is insufficient and blood pH increases (West, 2006). The rise in blood pH increases Hb-O_2_ affinity, counteracting the effects of an elevated 2,3-DPG production such that P_50_ approximates values observed at sea-level (Mairbaurl and Weber, 2012). As humans travel above ∼5400 m, Hb-O_2_ affinity increases as the respiratory alkalosis becomes more severe (West, 1984).

Contemporary studies suggest a potential role of hemoglobin found in cells other than erythrocytes such as alveolar epithelial cells, lung cells, and mesangial cells (Du et al., 2012; Saha et al., 2014). Within these non-erythrocytic cells, the production of hemoglobin appears to be upregulated in response to hypoxia (Cheung et al., 1997; Tezel et al., 2009; Grek et al., 2011), potentially serving as a “reservoir” for O_2_ (Saha et al., 2014). Therefore, a key area for future investigation is the relationship between non-erythroid hemoglobin production and hypoxia tolerance. However, there is currently minimal evidence to suggest that non-erythroid hemoglobin provides a functional impact on cardiovascular adjustments during hypoxia.

### Pharmacological Induction of High Hemoglobin-Oxygen Affinity

Several pharmacological methods which transfuse 2,3-DPG depleted red blood cells into both animals and humans have allowed investigation into the role of high Hb-O_2_ affinity in O_2_ transport (Riggs et al., 1973; Woodson et al., 1973; Wranne et al., 1974; Bakker et al., 1976; Malmberg et al., 1979; Woodson and Auerbach, 1982; Birchard and Tenney, 1991). However, methods used to achieve 2,3-DPG depletion often alter acid-base balance and total blood volume, potentially confounding the observed cardiorespiratory adjustments (Birchard and Tenney, 1991). More recent developments of pharmaceuticals that induce high Hb-O_2_ affinity allow examination of altered Hb-O_2_ affinity with fewer complications (Dufu et al., 2017; Kalfa et al., 2019; Stewart et al., 2020, 2021). For example, voxelotor binds allosterically to some, but not all hemoglobin and increases Hb-O_2_ affinity. Hemoglobin modified with voxelotor exhibits a reduced Bohr effect compared to unmodified hemoglobin (Pochron et al., 2019), which may limit O_2_ off-loading during instances where blood pH decreases such as rigorous exercise.

In general, allosteric modifiers allow for the manipulation of Hb-O_2_ affinity with less perturbations in acid-base balance associated with 2,3-DPG depletion techniques. However, in healthy humans voxelotor induces only a modest decrease in P_50_ of ∼2 mmHg (Stewart et al., 2020, 2021) compared to the greater range from 3 to 10 mmHg obtained *via* 2,3-DPG depletion (Gillette et al., 1974; Wranne et al., 1974). The ability to pharmacologically alter Hb-O_2_ affinity in humans both acutely and chronically may provide additional insights on the context-dependent circumstances at which high Hb-O_2_ affinity is advantageous (i.e., magnitude and duration of hypoxia).

### Humans With High Hemoglobin-Oxygen Affinity Hemoglobinopathies

Currently, over 200 distinct mutations resulting in high Hb-O_2_ affinity have been identified (Charache et al., 1966; Mangin, 2017). By definition, high Hb-O_2_ affinity is characterized by a P_50_ less than 24 mmHg (Figure 1; Rumi et al., 2009; Mangin, 2017). However, a majority of high Hb-O_2_ affinity hemoglobinopathies examined are associated with P_50_ values ranging from 12 to 17 mmHg (Table 1). Both the amino acid substitution and location at which the substitution occurs within the hemoglobin molecule may affect Hb-O_2_ affinity, cooperativity, and response to modulatory ligands. Within the hemoglobin mutations represented in this review (Table 1), all exhibit reduced cooperativity and only Hb Andrew-Minneapolis demonstrates a reduced Bohr effect (Adamson et al., 1969; Boyer et al., 1972; Nute et al., 1974; Zak et al., 1974; Wranne et al., 1983; Berlin et al., 2009). Lower cooperativity gives rise to the unique shape of the standard O_2_ dissociation curve in humans with high Hb-O_2_ affinity (Figure 1). However, the complex interactions between modulatory factors and subsequent effects on *in vivo* Hb-O_2_ affinity have not been clearly elucidated in mutated hemoglobin molecules.

Due to a lower P_50_, O_2_ off-loading is likely compromised in those with high Hb-O_2_ affinity. Evidence for compromised O_2_ off-loading may be seen through compensatory increases in hematocrit resulting in a higher O_2_ carrying capacity per unit of blood (Charache et al., 1966; Mangin, 2017; Shepherd et al., 2019). It is thought that the kidneys sense a reduction of O_2_ off-loading and promote red blood cell production in response, functioning as a “critmeter” (Donnelly, 2001). In addition to an elevated hematocrit humans with high Hb-O_2_ affinity likely develop skeletal muscle adaptations to compromised O_2_ off-loading such as a greater percentage of non-oxidative (type II) muscle fibers than their counterparts with normal Hb-O_2_ affinity (Wranne et al., 1983). Additionally, a greater accumulation of metabolic byproducts (e.g., lactate and H^+^) during high-intensity exercise have been reported in humans with high Hb-O_2_ affinity compared to those with normal Hb-O_2_ affinity (Länsimies et al., 1985; Dominelli et al., 2020). Those with high Hb-O_2_ affinity demonstrate a similar lactate accumulation at the end of exhaustive exercise during both normoxia and hypoxia, whereas controls demonstrate a reduced lactate accumulation during hypoxia compared to normoxia (Dominelli et al., 2020). A possible explanation for these observations may be that humans with high Hb-O_2_ affinity obtain similar power outputs in normoxia and hypoxia and therefore demonstrate a similar metabolite accumulation between the two conditions; whereas those with normal Hb-O_2_ affinity have a reduced power output and lower lactate concentrations during hypoxia compared to normoxia.

The observed differences in skeletal muscle fiber composition and utilization of metabolic pathways supporting exercise between humans with high Hb-O_2_ affinity and humans with normal Hb-O_2_ affinity may be due to differences in O_2_ off-loading kinetics and tissue P_*O*_2__ (Wranne et al., 1983). In general, many physiological compensatory responses coinciding with high Hb-O_2_ affinity remain uncharacterized. Key areas for future investigation include adaptations to high Hb-O_2_ affinity possibly affecting capillary density, blood flow distribution, and skeletal muscle aerobic capacity (Dempsey, 2020).

## High Hemoglobin-Oxygen Affinity and Cardiorespiratory Adjustments During Hypoxia at Rest

### Acute Hypoxia

**FIGURE 2:**
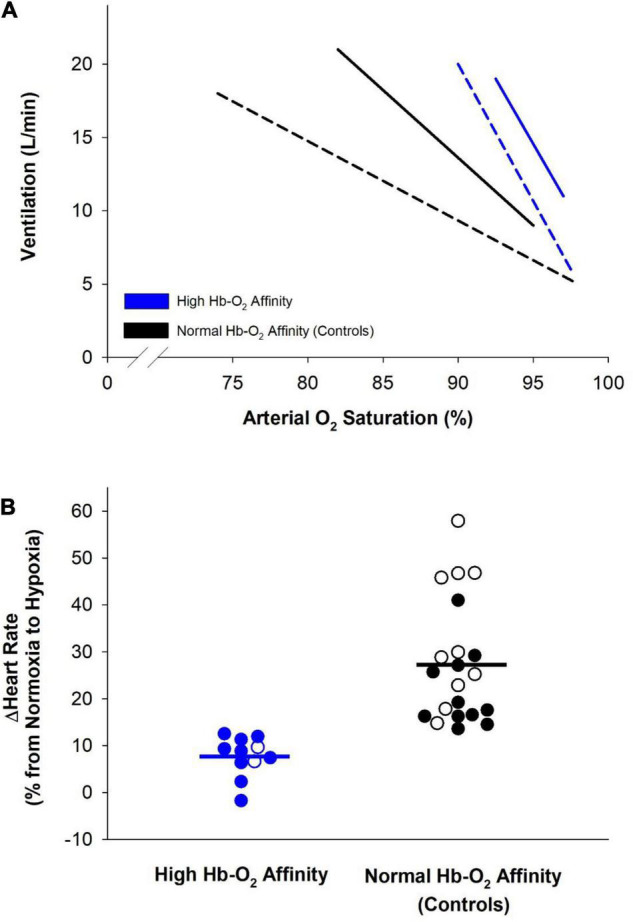
Cardiorespiratory adjustments elicited during hypoxia by humans with high hemoglobin-O_2_ (Hb-O_2_) affinity (blue lines and symbols) and controls with normal Hb-O_2_ affinity (black lines and symbols). **(A)** Relationship of minute ventilation and arterial O_2_ saturation among humans with high Hb-O_2_ affinity compared to normal Hb-O_2_ affinity controls during progressive isocapnic hypoxia. Dashed lines represent data from Hebbel et al. (1977) where hypoxia was increased such that alveolar P_*O*_2__ was lowered from 120 to 40 mmHg over ∼5 min (*n* = 2 humans with high Hb-O_2_ affinity and *n* = 2 humans with normal Hb-O_2_ affinity). Solid lines represent data from Dominelli et al. (2019) where hypoxia was increased such that end-tidal P_*O*_2__ was lowered from normal room-air values to 50 mmHg over ∼12 min (*n* = 9 humans with high Hb-O_2_ affinity and *n* = 12 humans with normal Hb-O_2_ affinity). **(B)** Percentage increase in heart rate during progression of normoxia to hypoxia among humans with high Hb-O_2_ affinity compared to normal Hb-O_2_ affinity controls. Open symbols represent data from Hebbel et al. (1977) where heart rate was compared at an alveolar P_*O*_2__ of 100 and 40 mmHg (*n* = 2 humans with high Hb-O_2_ affinity and *n* = 10 humans with normal Hb-O_2_ affinity). Filled symbols represent data from Dominelli et al. (2019) where heart rate was compared at normoxia and at an end-tidal P_*O*_2__ of 50 mmHg (*n* = 9 humans with high Hb-O_2_ affinity and *n* = 12 humans with normal Hb-O_2_ affinity). Solid bars represent the average change in heart rate in both groups.

Brief periods of hypoxia require both cardiovascular and respiratory adjustments to maintain adequate O_2_ delivery (Rowell and Blackmon, 1987; Bärtsch and Saltin, 2008; Naeije, 2010). One crucial immediate adjustment in response to hypoxia is increased ventilation which raises alveolar ventilation, increases arterial P_*O*_2__ and protects against arterial O_2_ desaturation (Otis et al., 1956; Dempsey and Forster, 1982). At a given alveolar P_*O*_2__, humans with high Hb-O_2_ affinity have similar minute ventilation compared to humans with normal Hb-O_2_ affinity (Hebbel et al., 1977; Rossoff et al., 1980; Dominelli et al., 2019). Yet, due to the left-shifted nature of their oxygen dissociation curve, those with high Hb-O_2_ affinity have a higher arterial O_2_ saturation at a given alveolar P_*O*_2__ (Figure 2A; Hebbel et al., 1977; Rossoff et al., 1980; Dominelli et al., 2019).

In addition to increased ventilation, hypoxia is associated with increased cardiac output, primarily through an elevated heart rate (Brown and Grocott, 2013; Siebenmann and Lundby, 2015). As arterial O_2_ saturation decreases during hypoxia, cardiac output increases and peripheral arterioles dilate to match O_2_ delivery and demand (Ekblom et al., 1975; Phillips et al., 1988). These observations suggest that the change in heart rate during acute hypoxia is closely linked to systemic O_2_ delivery (Casey and Joyner, 2011; Joyner and Casey, 2014; Siebenmann and Lundby, 2015). During acute hypoxia, humans with high Hb-O_2_ affinity display a lesser increase in heart rate, and presumably cardiac output, likely due to better maintained arterial O_2_ content (Figure 2B; Hebbel et al., 1977; Dominelli et al., 2019). Since arterial O_2_ saturation remains fairly constant in humans with high Hb-O_2_ affinity during modest reductions of P_*O*_2__, as occurs at moderately high altitude, arterial O_2_ content is better maintained and heart rate increases to a lesser extent compared to those with normal Hb-O_2_ affinity.

Peripheral chemosensors located at both the carotid and aortic bodies respond to acute changes in arterial P_*O*_2__ and P_*CO*_2__, such as during normobaric and hypobaric hypoxia (Lahiri and Forster, 2003). Stimulation of peripheral chemosensors during hypoxic exposure causes an increase in minute ventilation and sympathetic activity in an attempt to maintain O_2_ homeostasis (Powell et al., 1998; Bernardi et al., 2001; Fletcher, 2001). Examination of humans with high Hb-O_2_ affinity provides support for low P_*O*_2__ being a strong stimulus in the hypoxic ventilatory response, rather than arterial O_2_ saturation or content (Hebbel et al., 1977; Rossoff et al., 1980; Dominelli et al., 2019). Some evidence suggests that aortic chemosensors sense changes in arterial O_2_ content and heart rate is adjusted accordingly (Lugliani et al., 1971; Wasserman, 1978; Lahiri et al., 1980, 1981). Therefore, the lower heart rate during hypoxia among humans with high Hb-O_2_ affinity compared to controls may be caused by decreased sensory stimulus of the aortic chemosensors (Dominelli et al., 2019). However, the mechanistic stimulation of the peripheral chemosensors requires that O_2_ be dissociated from hemoglobin to be sensed (Lopez-Barneo et al., 2001). Therefore, the relationship between O_2_ content and P_*O*_2__ sensed at the carotid chemosensors remains unclear and contention exists regarding mechanisms of O_2_ sensing and regulation of systemic blood flow (Ward, 2008). Detailed discussions into the mechanism of O_2_ sensing are provided elsewhere (Lopez-Barneo et al., 2001; Kumar and Prabhakar, 2012).

The observed relationship between ventilation and arterial O_2_ saturation may present a disadvantage to humans with high Hb-O_2_ affinity during acute hypoxic exposure. Since the stimulus for ventilation is closely linked to arterial P_*O*_2__ and not arterial O_2_ saturation during brief periods of hypoxia (Biscoe, 1971; Guz, 1975; Weil and Zwillich, 1976), humans with high Hb-O_2_ affinity have an excessive ventilatory response despite only a modest drop in arterial O_2_ saturation and delivery (Figure 2A). Excessive ventilation increases O_2_ consumption by respiratory muscles (Cherniack, 1959; Robertson et al., 1977). Although accounting for a small percentage of total O_2_ consumption during rest, respiratory muscle O_2_ demand increases during hyperventilation or exercise (Aaron et al., 1992; Coast et al., 1993; Dominelli et al., 2015). Thus, during exercise, there are increased and competitive demands for O_2_ in metabolically active tissue including both exercising muscle and respiratory muscle (Harms et al., 2000; Sheel et al., 2001; Romer and Polkey, 2008; Dominelli et al., 2017). This competition for blood flow between respiratory and exercising muscle limits exercise tolerance at higher and extreme altitudes and is often referred to as “respiratory steal” (Pugh et al., 1964; Schoene, 2001; Helfer et al., 2016). The physiological consequences of “respiratory steal” are likely exacerbated at more extreme altitudes as hyperventilation, and thus metabolic demand of respiratory muscle, becomes more pronounced. Therefore, the excessive hyperventilation during acute hypoxic exposure may be disadvantageous for humans with high Hb-O_2_ affinity due to increased O_2_ consumption by respiratory muscles with minimal improvement in arterial O_2_ saturation.

### Chronic Hypoxia

In addition to acute hypoxic exposure, the benefits of high Hb-O_2_ affinity have been observed through examination of cardiorespiratory adjustments during 10-days of residing at high altitude (Leadville, Colorado, ∼3100 m elevation) (Hebbel et al., 1978). Two humans with high Hb-O_2_ affinity and two of their siblings with normal Hb-O_2_ affinity were examined during the acclimatization period. Changes in arterial 2,3-DPG concentration and pH were similar during the stay at high altitude in both sets of siblings. However, peak and average heart rate during acclimatization were lower in the siblings with high Hb-O_2_ affinity. During hypoxia, impaired O_2_ delivery to the kidneys prompts erythropoietin production (Donnelly, 2001; Nangaku and Eckardt, 2007; Haase, 2013). Erythropoietin stimulates red blood cell production and leads to a subsequent increase in O_2_ carrying capacity to compensate for impaired O_2_ delivery (Erslev, 1991; Jelkmann, 2011). Humans with high Hb-O_2_ affinity showed smaller increases in erythropoietin production when residing at high altitude (Hebbel et al., 1978). A lesser erythropoietin production during high-altitude acclimatization suggests that O_2_ delivery is better preserved among humans with high Hb-O_2_ affinity. Similarly, Hall et al. (1936) showed that mammals native to high altitude display a reduced erythropoietic response during travel from low altitude to high altitude. Combined, these findings suggest that lessened cardiovascular adjustments are needed to maintain adequate O_2_ delivery during high-altitude acclimatization in humans with high Hb-O_2_ affinity compared to those with normal Hb-O_2_ affinity.

Marked physiological compensations are required to maintain homeostasis during sojourn to extreme altitudes (West, 2006). Hb-O_2_ affinity increases at altitudes greater than ∼5400 m due to severe respiratory alkalosis with insufficient renal compensations (see section “Hemoglobin-Oxygen Affinity”). During ascent to the summit of Mt. Everest, ∼8100 m, climbers had a reduction in P_50_ from ∼26 mmHg to less than ∼20 mmHg (West, 1984). A more recent study examining blood oxygenation of four climbers reported arterial saturations ranging from 34 to 70% at the summit of Everest (Grocott et al., 2009). Without an increase of Hb-O_2_ affinity due to respiratory alkalosis it is likely that humans would not be able to reach the summit without supplemental O_2_.

As extreme altitude challenges the ability to transport O_2_ from atmospheric air to tissue, the modulation of Hb-O_2_ affinity is crucial to maintain adequate O_2_ consumption. Enhanced O_2_ loading in the lungs due to high Hb-O_2_ affinity is even more advantageous at extreme altitude than at high altitude, where ambient P_*O*_2__ can fall to as low as 40 mmHg, outweighing potential limitations in O_2_ off-loading (Eaton et al., 1974). The ventilatory response during hypoxia is similar between humans with genetic mutations leading to high Hb-O_2_ affinity and those with normal Hb-O_2_ affinity (Hebbel et al., 1977; Rossoff et al., 1980; Dominelli et al., 2019). Under the circumstances of extreme altitude, humans with high Hb-O_2_ affinity may develop respiratory alkalosis to a similar degree as observed in humans with normal Hb-O_2_ affinity (West, 1984; Grocott et al., 2009). In addition, some genetic hemoglobin mutations demonstrate a preserved Bohr effect, such that Hb-O_2_ affinity would decrease during respiratory alkalosis by a similar magnitude compared to non-mutated hemoglobin (Adamson et al., 1969; Boyer et al., 1972; Nute et al., 1974; Wranne et al., 1983; Berlin et al., 2009). A physiological consequence of respiratory alkalosis would be further left-shifted O_2_ dissociation curve adding additional protection against arterial desaturation. Therefore, humans with genetic modifications resulting in high Hb-O_2_ affinity and a preserved Bohr effect may ascend to extreme altitudes with fewer physiological complications (i.e., Acute mountain sickness, high-altitude cerebral edema, and impaired cognitive function) compared to sojourners with normal Hb-O_2_ affinity. However, to our knowledge no humans with genetic high Hb-O_2_ affinity have been examined at altitudes greater than ∼3100 m and the proposed physiologic responses to higher and extreme altitudes are theoretical.

Groups of indigenous humans who have resided at high altitude for many generations display genotypic and phenotypic adaptations to the hypoxic environment (Beall, 2007, 2014; Moore, 2017; Tymko et al., 2019; Storz, 2021). Recent evidence has suggested an adaptive increase of Hb-O_2_ affinity among high altitude natives of the Qinghai-Tibetan Plateau (>3500 m) compared to sea-level residents (Simonson et al., 2014; Li et al., 2018). However, others have reported that some high-altitude populations [Nepalese (>3800 m), Peruvian (>4500 m), and Qinghai-Tibetan (>3500 m) natives] do not show this adaptive increase in Hb-O_2_ affinity (Samaja et al., 1979; Winslow et al., 1981; Tashi et al., 2014). Additional studies may improve understanding of changes in Hb-O_2_ affinity observed among high-altitude natives and molecular mechanisms underlying such adaptation.

The Qinghai-Tibetan natives had a P_50_ ∼2 mmHg lower than the sea-level residents (24.5 vs. 26.2 mmHg, respectively) (Simonson et al., 2014). However, the high-altitude natives did not display improvements in pulmonary gas exchange or peak exercise capacity during hypoxia compared to the sea-level residents, suggesting no clear benefit of high Hb-O_2_ affinity in the population examined. These findings, contradictory to those observed in humans with genetic mutations resulting in high Hb-O_2_ affinity, could be explained by differences in the magnitude of P_50_. The high-altitude natives studied had a P_50_ of ∼25 mmHg, in contrast to values ranging from 12 to 17 mmHg observed in humans with genetic mutations resulting in high Hb-O_2_ affinity (Table 1). Therefore, the P_50_ observed in the high-altitude native population is probably not low enough to warrant significant alterations in pulmonary gas exchange, O_2_ extraction, and exercise capacity during hypoxia. In addition, adaptations of high-altitude populations, which affect multiple steps within the O_2_ transport cascade (Beall, 2007), may confound our ability to clearly dissociate the role of increased Hb-O_2_ affinity in humans native to high altitude.

## High Hemoglobin-Oxygen Affinity and Cardiorespiratory Adjustments During Exercise

### Maximal Oxygen Consumption During Normoxia

Studies examining the effects of pharmacologically induced high Hb-O_2_ affinity on O_2_ consumption during normoxia have provided discordant results in both humans and animals (Riggs et al., 1973; Woodson et al., 1973; Wranne et al., 1974; Valeri et al., 1975; Yhap et al., 1975; Bakker et al., 1976; Malmberg et al., 1979; Ross and Hlastala, 1981; Woodson and Auerbach, 1982; Stewart et al., 2020, 2021). Recently, Stewart et al. (2021) showed that pharmaceutical induction of high Hb-O_2_ affinity (only ∼2 mmHg decrease in P_50_) using voxelotor reduced normoxic maximal O_2_ consumption ($\dot{\text{V}}$O_2max_) in humans. The decrement in normoxic $\dot{\text{V}}$O_2max_ observed by Stewart et al. (2021) could be due to both an increase in Hb-O_2_ affinity and a reduced Bohr effect: the transient reduction of Hb-O_2_ affinity with decreasing pH (Pochron et al., 2019). A reduced Bohr effect in exercising muscle would further compromise O_2_ off-loading, particularly during periods of high metabolic demand (Mairbäurl, 2013). Conversely, some mathematical models suggest that normoxic $\dot{\text{V}}$O_2max_ is relatively insensitive to modest increases in Hb-O_2_ affinity despite limitations in O_2_ off-loading (Wagner, 1997; Shepherd et al., 2019).

In corroboration with results found through mathematical modeling, humans with high Hb-O_2_ affinity show no difference in normoxic $\dot{\text{V}}$O_2max_ values compared to similar age, sex-matched controls with normal Hb-O_2_ affinity (Länsimies et al., 1985; Dominelli et al., 2020). However, there is evidence for altered metabolic processes among humans with high Hb-O_2_ affinity compared to controls during exercise testing. During cycling exercise in normoxia, humans with high Hb-O_2_ affinity may have greater reliance on anaerobic metabolism during heavy to maximal exercise, as evidenced by lower blood pH and pronounced lactate production compared to controls (Wranne et al., 1983; Länsimies et al., 1985; Dominelli et al., 2020). In addition, humans with high Hb-O_2_ affinity seem to display a worsened exercise efficiency during cycling, i.e., higher O_2_ consumption for a given power output (Dominelli et al., 2020). Theoretically, compromised O_2_ off-loading due to high Hb-O_2_ affinity may give rise to the greater reliance on anaerobic metabolism, which contributes to the worsened exercise efficiency observed (Dominelli et al., 2020). In brief, current evidence indicates that humans with high Hb-O_2_ affinity have similar normoxic $\dot{\text{V}}$O_2max_ values despite altered metabolic processes during high-intensity exercise.

Little is known about the relationship between high Hb-O_2_ affinity and compensatory mechanisms that facilitate adequate O_2_ extraction. Wranne et al. (1983) demonstrated that the arterial-venous O_2_ extraction was abnormally low during exercise in humans with high Hb-O_2_ affinity, suggesting that O_2_ off-loading may be compromised within muscle during whole-body exercise. However, humans with high Hb-O_2_ affinity had a ∼25% greater O_2_ carrying capacity than those with normal Hb-O_2_ affinity, likely compensating for the diminished arterial-venous O_2_ extraction both at rest and during exercise. The potential benefits of high Hb-O_2_ affinity are likely contingent on the capacity to extract O_2_ from blood (Wearing et al., 2021). The capacity of O_2_ off-loading and diffusion to the mitochondria are crucial to maximize O_2_ utilization in cases of high Hb-O_2_ affinity, especially during peak whole-body exercise. Therefore, future research should focus on the relationship between high Hb-O_2_ affinity and compensatory mechanisms which facilitate adequate O_2_ extraction within peripheral tissue such as alterations in the microvascular architecture, flow of the red blood cells through the microvasculature, and the diffusion gradients driving O_2_ to the mitochondria.

### Maximal Oxygen Consumption During Hypoxia

**FIGURE 3:**
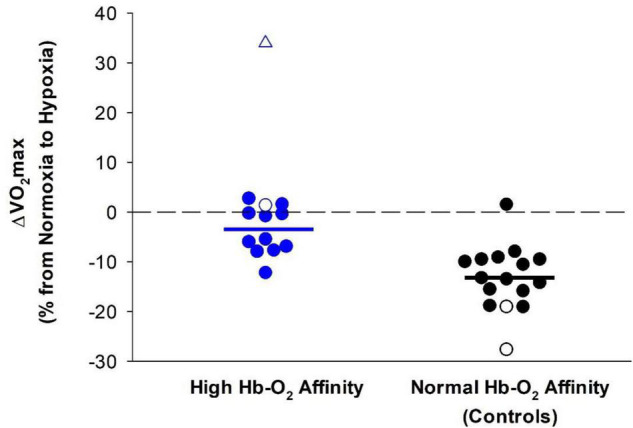
Difference in $\dot{\text{V}}$O_2max_ between normoxia and hypoxia in humans with high Hb-O_2_ affinity (blue symbols) (–4 ± 5% without outlier) compared to normal Hb-O_2_ affinity controls (black symbols) (–13 ± 6%). Open symbols represent data from Hebbel et al. (1978). The open triangle represents an outlier not included in the calculation of the mean. Closed symbols represent data from Dominelli et al. (2020). Solid bars represent the average change in $\dot{\text{V}}$O_2max_ in both groups not including the outlier with high Hb-O_2_ affinity. The dashed line provides a reference for no change.

Maximal O_2_ consumption in humans decreases with increasing severity of hypoxia (Faulkner et al., 1968; Grover, 1970; Lawler et al., 1988; Ferretti et al., 1997; Wehrlin and Hallén, 2006; Wagner, 2010; West, 2010). However, humans with high Hb-O_2_ affinity are better able to maintain $\dot{\text{V}}$O_2max_ during hypoxia compared to those with normal Hb-O_2_ affinity (Figure 3). As previously described, Hebbel and colleagues examined four siblings, two with high Hb-O_2_ affinity and two with normal Hb-O_2_ affinity, during 10 days of high-altitude acclimatization (Leadville, Colorado, ∼3100 m elevation). At high altitude $\dot{\text{V}}$O_2max_ decreased by ∼28 and 19% compared to sea-level values in the two siblings with normal Hb-O_2_ affinity (Hebbel et al., 1978). On the other hand, the two siblings with high Hb-O_2_ affinity did *not* demonstrate a reduction in $\dot{\text{V}}$O_2max_ at high altitude compared to low altitude (Hebbel et al., 1978).

Similarly, experiments using acute normobaric hypoxia showed that humans with high Hb-O_2_ affinity had better maintained $\dot{\text{V}}$O_2max_ during hypoxia compared to humans with normal Hb-O_2_ affinity (Dominelli et al., 2020). In addition, peak power output during cycling exercise was better preserved in those with high Hb-O_2_ affinity (Dominelli et al., 2020). At both high altitude and normobaric hypoxia, there was no difference in maximal heart rate during exercise in humans with high Hb-O_2_ affinity compared to those with normal Hb-O_2_ affinity (Hebbel et al., 1978; Dominelli et al., 2020). Previous studies indicate that an increase in blood viscosity associated with an elevated hematocrit, common in humans with chronic high Hb-O_2_ affinity, may limit blood flow and maximal cardiac output in humans (Richardson and Guyton, 1959; Schumacker et al., 1985; Çınar et al., 1999). On the contrary, some studies suggest that systemic blood flow at rest and during exercise within animals is not reduced at a hematocrit of ∼50–60% (Gaehtgens et al., 1979; Schumacker et al., 1985; Lindenfeld et al., 2005). However, hematocrits greater than 60% likely result in a substantially elevated blood viscosity such that systemic blood flow is restricted (Weisse et al., 1964; Gaehtgens et al., 1979; Schumacker et al., 1985). Therefore, it is unclear whether cardiac output and systemic blood flow is limited among humans with high Hb-O_2_ affinity where hematocrit often ranges from ∼55 to 65%.

The reduction of $\dot{\text{V}}$O_2max_ during hypoxia is directly related to the degree of arterial desaturation (Hughes et al., 1968; Calbet et al., 2003a). As such, a higher arterial O_2_ saturation in humans with high Hb-O_2_ affinity for a given level of hypoxia likely contributes to the preservation of hypoxic $\dot{\text{V}}$O_2max_ (Figure 3). In humans with normal Hb-O_2_ affinity at high altitude, hypoxic $\dot{\text{V}}$O_2max_ is less than values measured at sea-level and hypoxic $\dot{\text{V}}$O_2max_ either remains the same or progressively increases during acclimatization (Saltin et al., 1968; Calbet et al., 2003b). Despite acclimatization, hypoxic $\dot{\text{V}}$O_2max_ does not reach values previously measured at sea-level (Calbet et al., 2003b). In contrast, humans with high Hb-O_2_ affinity have a better maintained hypoxic $\dot{\text{V}}$O_2max_ upon transition to high altitude, but it is unknown how humans with high Hb-O_2_ affinity may acclimatize to high altitude and subsequent effects on hypoxic $\dot{\text{V}}$O_2max_.

## Conclusion

High Hb-O_2_ affinity has been identified as a potentially advantageous adaptation to high altitude in several animal species. From a cardiorespiratory perspective, we suggest that high Hb-O_2_ affinity is advantageous for humans when exposed to hypoxic environments both at rest and during exercise. During hypoxia, humans with high Hb-O_2_ affinity exhibit lessened increases in heart rate, reduced erythropoietin production, and higher arterial O_2_ saturation at rest compared to those with normal Hb-O_2_ affinity. In addition, $\dot{\text{V}}$O_2max_ and work capacity are better maintained during hypoxia compared to normoxia in humans with high Hb-O_2_ affinity. The advantages associated with high Hb-O_2_ affinity are likely potentiated as the degree of hypoxia becomes more severe. In addition, high Hb-O_2_ affinity confers physiological disadvantages at less severe magnitudes of hypoxia such as reduced O_2_ off-loading and unwarranted hyperventilation when arterial O_2_ saturation is fairly well-preserved. However, current understanding on the effects of high Hb-O_2_ affinity during hypoxia is largely limited to normobaric hypoxia. Future research warrants the investigation into the influence of high Hb-O_2_ affinity during both short- and long-term periods of high-altitude acclimatization. In addition, long-term adaptations to pharmaceutically induced high Hb-O_2_ affinity in humans remains largely unexamined. Regardless, the influence of high Hb-O_2_ affinity on cardiorespiratory adjustments to environmental hypoxia is of key interest in human adaptation to environmental hypoxia, particularly during bouts of exercise.

## Author Contributions

MJ and CW conceived the concept for this review. KW, JS, and CW drafted the manuscript. PD, SB, JS, SK, and MJ provided critical revision of the manuscript for important intellectual content. All authors approved the final version of the manuscript.

## Conflict of Interest

The authors declare that the research was conducted in the absence of any commercial or financial relationships that could be construed as a potential conflict of interest.

## Publisher’s Note

All claims expressed in this article are solely those of the authors and do not necessarily represent those of their affiliated organizations, or those of the publisher, the editors and the reviewers. Any product that may be evaluated in this article, or claim that may be made by its manufacturer, is not guaranteed or endorsed by the publisher.
